# Mechanism of Action of Acupuncture in Obesity: A Perspective From the Hypothalamus

**DOI:** 10.3389/fendo.2021.632324

**Published:** 2021-04-02

**Authors:** Li Wang, Chao-Chao Yu, Jia Li, Qing Tian, Yan-Jun Du

**Affiliations:** ^1^ College of Acupuncture and Orthopedics, Hubei University of Chinese Medicine, Wuhan, China; ^2^ Department of Tuina, Shenzhen Traditional Chinese Medicine Hospital, Shenzhen, China; ^3^ The Fourth Clinical College, Guangzhou University of Chinese Medicine, Shenzhen, China; ^4^ Department of Pathology and Pathophysiology, School of Basic Medicine, Tongji Medical College, Huazhong University of Science and Technology, Wuhan, China

**Keywords:** acupuncture, obesity, hypothalamus, mechanism, action

## Abstract

Obesity is a prevalent metabolic disease caused by an imbalance in food intake and energy expenditure. Although acupuncture is widely used in the treatment of obesity in a clinical setting, its mechanism has not been adequately elucidated. As the key pivot of appetite signals, the hypothalamus receives afferent and efferent signals from the brainstem and peripheral tissue, leading to the formation of a complex appetite regulation circuit, thereby effectively regulating food intake and energy homeostasis. This review mainly discusses the relationship between the hypothalamic nuclei, related neuropeptides, brainstem, peripheral signals, and obesity, as well as mechanisms of acupuncture on obesity from the perspective of the hypothalamus, exploring the current evidence and therapeutic targets for mechanism of action of acupuncture in obesity.

## Introduction

Obesity is a condition that is exceedingly prevalent worldwide and has been considered a risk factor for hypertension, diabetes, hyperlipidemia, fatty liver, cardiovascular disease, and sleep apnea (1). It not only increases the expenses associated with healthcare but also greatly reduces the wellness-related quality of life. More than 2 billion adults worldwide are overweight or obese and the prevalence of obesity continues to increase globally, thus the World Health Organization came up with the term of “globesity” (2). Currently, obesity has reached the state of a global epidemic and is a serious cause of concern.

Obesity does not stem from a simple problem of self-control, rather it occurs owing to the chronic, excessive accumulation of body-fat, caused by long-term disorders in energy metabolism and appetite regulation (3, 4). The extent of obesity is usually measured using the body mass index (BMI). Its etiology is a complex interaction that includes genetic predisposition, individual behavior, and environmental factors (5). Although several studies on obesity have been conducted, the potential mechanisms underlying this disorder remain unclear. Conventional therapies to treat obesity include dietary restriction, physical exercise, and bariatric surgery (6, 7). Lifestyle modification is often always the first line of treatment for obesity; however, the resultant weight loss following this approach is minimal. Furthermore, studies on diet and behavioral therapy have shown that it is difficult to maintain weight stability when obesity is associated with various metabolic changes (8). Owing to the differences in individual lifestyles and a lack of uniform standards, achieving adequate weight control still poses a problem. Only a few anti-obesity drugs have been approved by the FDA for the long-term management of obesity; however, these drugs are associated with gastrointestinal adverse side effects, such as vomiting, nausea, diarrhea, and other side effects (9), which greatly limit their use. Thus, complementary and alternative therapies are being pursued to overcome these limitations. Existing evidence-based reviews have demonstrated that acupuncture is efficacious in the treatment of obesity (10, 11). Acupuncture, one of the oldest medical therapies and as a crucial segment of traditional Chinese medicine (TCM), has been widely used to treat various diseases for at least 3000 years. Meanwhile, it is also one of the most popular complementary and alternative approaches, which is endorsed by the WHO and the National Institutes of Health (NIH) (12–14). It is a relatively safe and inexpensive therapy in which stainless steel needles are inserted into specific acupoints to achieve the sensation of Deqi and produce therapeutic effects. The treatment efficacy is further enhanced by electrical stimulation or manual manipulation. Electroacupuncture (EA) is a technique that has evolved over the years and involves the conduction of electrical stimuli using acupuncture needles at certain frequencies. The parameters of EA, such as waveforms, frequencies, intensity, and stimulus time, can be accurately characterized. It is reported that acupuncture has positive effects on the central nervous system (15), metabolism (16), gastrointestinal system (17), and immune system (18), and in the treatment of obesity. Acupuncture may bring about weight loss by regulating appetite and energy expenditure (19). However, the specific mechanism of acupuncture on obesity is still a new field that needs further exploration.

Recent evidence suggests that the experimental mechanism of acupuncture on obesity is mainly focused on the central nervous system (CNS) and peripheral adipose tissue (20). The CNS is involved in multiple pathways and plays an important role in maintaining energy homeostasis and responding to various signals. These signals are integrated by the peripheral nerves, which modulate the central neuropeptides *via* the regulation of food intake and energy homeostasis (21). Despite several regions of the brain being involved in this complex process, most studies have concentrated on the hypothalamus. It is now being increasingly recognized that the expression of appetite is chemically encoded in the hypothalamus (22). The hypothalamus not only constitutes a crucial brain area modulating food intake and energy homeostasis *via* neural and hormonal signals but also plays a significant role in the beneficial effects of acupuncture on obesity (23). The hypothalamus transmits neuronal-pathway signals through hypothalamic nuclei and related neuropeptides, interacting with the brain stem and peripheral signals, thereby regulating food intake and energy balance and ultimately achieving the goal of weight control. In this review, we probe into the interplay between the hypothalamic nuclei, related neuropeptides, brainstem, peripheral signals, and obesity, mainly derived from studies in rat models, and explore the mechanism of acupuncture on obesity from the perspective of the hypothalamus, and identify potential new targets for acupuncture in the treatment of obesity.

## Hypothalamic Nuclei and Related Neuropeptides

The hypothalamus is divided into several interconnected nuclei, mainly including the arcuate nucleus (ARC), ventromedial hypothalamic nucleus (VMH), paraventricular nucleus (PVN), dorsomedial hypothalamic nucleus (DMH), and lateral hypothalamic area (LHA) (24). The PVN, DMH, VMH, LHA, and peripheral zones receive neuronal projections of neuropeptide Y (NPY)/agouti gene-related protein (AgRP) and pro-opiomelanocortin (POMC)/cocaine- and amphetamine-regulated transcript (CART) from the ARC (25). Various neuronal pathways between these nuclei are organized into complex regulatory networks in which some neuropeptides are released to affect the long-term regulation of appetite and energy metabolism.

## Arcuate Nucleus (ARC)

The arcuate nucleus (ARC) is situated at the mediobasal hypothalamus, adjacent to the third ventricle and lies close to the median eminence. Owing to the semipermeable blood–brain barrier, it receives metabolic signals directly from the peripheral circulation of the cerebrospinal fluid. The ARC is identified as a vital region for the mediation of energy homeostasis, which integrates circulating peripheral signals and encodes signals related closely to energy into the synaptic transmission, thereby affecting downstream pathways. It consists of at least two distinct neuronal populations with opposing effects in appetite regulation. One of them expresses two orexigenic polypeptides, namely, NPY and AgRP, whereas the other expresses the anorexigenic polypeptides, POMC and CART (26). Neuronal projections from these two populations not only extensively interact with other hypothalamic regions containing the PVN, DMH, and LHA (27), but are also influenced by related peripheral hormones, such as leptin, insulin, and ghrelin. Both NPY/AgRP and POMC/CART neurons are the primary neurons of peripheral metabolic signals, which play a part by binding to distinct sets of receptors, such as leptin, insulin, and ghrelin.

## Regulation of NPY/AgRP NEURONS

NPY or neuropeptide Y, a 36 amino acid peptide, belonging to the pancreatic polypeptide family, is abundant in the neurons in the central and peripheral nervous systems (28). In the hypothalamus, the NPY neurons are mainly distributed in the ARC and DMH, and the axons of the nerve fibers are chiefly projected into the PVN and LHA. NPY is one of the major hypothalamic neurons that senses and integrates peripheral energy signals from hormones and the diet. The level of NPY in the hypothalamus reflects the nutritional state of the body, an important characteristic of the long-term regulator of the energy balance (29). Levels of NPY and NPY mRNA increase during the fasting state and decrease after refeeding (30, 31). Studies report that repeated intracerebroventricular (ICV) injection of NPY leads to hyperphagia and obesity (32, 33). NPY acts on five different receptors (Y1–Y5 receptors) and functions *via* diverse mechanisms to arouse appetite; studies have shown that the Y1 and Y5 receptors of NPY mainly exert orexigenic effects and are involved in energy metabolism (34). Evidence indicates that NPY neuron is a primary feeding center to induce food intake in response to peripheral metabolic state (35).

Kim et al. (36) observed the effect of auricular acupuncture on the expression of NPY in the hypothalamic ARC of food-deprived rats. After auricular acupuncture, the levels of NPY significantly decreased in food-deprived rats. Auricular acupuncture combined with food restriction (FR) can effectively reduce the expression of NPY and appears to be an effective method. However, this research group also showed that acupuncture at Zusanli (ST36) enhanced the expression of NPY in diabetic rats (37). Taking the FR factors into account, Tian et al. (38) further studied the effect of EA on NPY expression, in which rats in the FR group were allowed access to food for merely one hour per day. Under this circumstance, EA obviously reduced food intake and body weight and was accompanied by a decline of NPY mRNA expression in FR rats. Similar findings in obese rats have also been reported by Shu (39). The inconsistent results might due to the dual effects of acupuncture, which led to differences in the expression of NPY in different experimental models. Taken together, these findings support that acupuncture can reduce NPY levels and food intake in rodent models, thereby achieving the goal of weight loss.

AgRP is a peptide hormone produced by hair follicle cells, which inhibits the production of melanin by the α-melanocyte-stimulating hormone (α-MSH). AgRP is uniquely expressed in the ARC and co-expressed with most NPY neurons and is an endogenous antagonist of the melanocortin-3 and melanocortin-4 receptors (MC3R and MC4R). Rossi and colleagues (40) have reported that like NPY, and AgRP is also an orexigenic peptide when injected *via* the ICV route. It has also been found that the release of AgRP increases in the hypothalamus during fasting (31). Furthermore, the excessive expression of AgRP in mice results in obesity (41). Consequently, the interaction between NPY and AgRP neurons makes it possible to regulate appetite and metabolism, with activation of both NPY receptors and antagonism of MC3R and MC4R by AgRP.

Liu et al. (42) explored the mechanism of EA in the treatment of obese rats. EA at ST36 and Quchi (LI11) inhibits the expression of AgRP and NPY and improves insulin resistance to achieve weight control. ST36 is the uniting point of the stomach channel of foot Yang Ming, which has the effect of regulating the stomach and intestines. Acupuncture on ST36 may enhance gastrointestinal motility and promote digestion. Moreover, Ren et al. (43) have reported that EA can upregulate the expression of POMC and downregulate that of AgRP and reduce appetite and food intake, thus effectively controlling body weight of obese rats. In summary, these findings support that acupuncture can reduce body weight by lowering the expression of AgRP in obese rats.

## Regulation of POMC/CART Neurons

POMC is a common precursor molecule of various neuropeptides, including α-MSH. The majority of the POMC neurons is located in the ARC and is projected onto the brainstem, NPY neurons, and PVN. Cleavage of POMC gives rise to several peptides called melanocortins, which function by binding to the melanocortin receptors (MC-Rs) coupled to the G proteins. The melanocortin neuropeptide, α-MSH, is extensively distributed in the hypothalamus, which binds to downstream MC4-Rs and leads to the inhibition of food intake (44). According to these findings, POMC knockout in mice has been reported to result in hyperphagia and weight gain (45). Also, POMC neurons in the ARC have been demonstrated to exert crucial actions on energy homeostasis *via* integrating signals from peripheral hormones, such as leptin and insulin (46).

Previous studies by Tian et al. (47, 48) indicate that the expression of POMC and α-MSH significantly decreased in obese rats, while EA treatment of bilateral ST36 and Sanyinjiao (SP6) upregulated the expression of POMC and α-MSH in the ARC of the hypothalamus, which inhibited food intake and ultimately led to weight loss in obese mice. Similar findings in obese rats have been reported by Wang and Ji (49, 50). Huang et al. have reported that 2 Hz EA at ST36, Guanyuan (CV4), Zhongwan (CV12) and Fenglong (ST40) can reduce food intake, glucose and lipid metabolism and body weight of obese rats, likely *via* promoting the expression of SIRT1 and POMC in the hypothalamus (51). Moreover, Shu et al. (39) further investigated the mechanism of EA on obese rats from the perspective of histone acetylation modification; their findings confirm that EA upregulates the expression of hypothalamic SIRT1 and downregulates the acetylation of FOXO1 in ARC, and increases the expression of POMC, thus inhibiting food intake and promoting weight reduction. And epigenetics (such as DNA methylation, histone acetylation, noncoding RNAs) may provide new insights for revealing the effects of acupuncture on obesity. Taken together, these findings support that acupuncture can suppress appetite and alleviate obesity by upregulating the expression of POMC in obese animal models.

POMC neurons and CART are co-expressed in the ARC. CART is a major anorexigenic peptide expressed in the hypothalamus. It is abundant in the ARC and PVN, and plays a role in activating the hypothalamic and brainstem neurons that are correlated with the central control of feeding behavior and energy metabolism (52, 53). CART-knockout mice showed an increase in body weight (54), while the ICV administration of CART inhibited food intake and weight gain (55). Tian et al. (56) reported that EA stimulation at ST36 and SP6 can effectively reverse the low expression of CART peptide to approximately normal levels in the ARC, resulting in the suppression of food intake and weight reduction in obese rats. However, few studies have investigated the effects of acupuncture on CART, and its definite effect on the regulation of CART in obese models should be investigated in future studies.

Leng et al. (57) have reported that EA at ST36, SP6, CV12 and Tianshu (ST25) may decrease methylation of Tsc1 and suppress the activity of mTORC1, reduce the expression of NPY and AgRP, and promote the expression of POMC, thereby regulating appetite and alleviating obesity. From the perspective of epigenetics, it also suggested that acupuncture may simultaneously exert actions on the regulation of orexigenic/anorexic peptides in the hypothalamic ARC, thereby regulating food intake and mitigating obesity. In summary, these findings support that the upregulated expression of POMC and α-MSH, and the downregulated expression of NPY and AgRP in the hypothalamic ARC, may constitute the main mechanisms underlying the effect of acupuncture on the reduction in food intake and body weight. Mechanisms of action of acupuncture on CART remains to be verified.

## Paraventricular Nucleus (PVN)

The PVN, located at the top of the third ventricle in front of the hypothalamus, constitutes pivotal second-order neurons downstream of the ARC. It integrates the projections of melanocortin, AgRP and NPY from the ARC and other signals from multiple sites in the brain. It is also sensitive to many neuropeptides related to appetite regulation (58). ICV administration of melanocortin agonists into the PVN leads to the inhibition of food intake, while the administration of melanocortin antagonists results in a notable increase in food intake and subsequent weight gain (59, 60). Corticotrophin-releasing hormone (CRH), an anorexigenic peptide, is synthesized in the subcells of the PVN and acts as an integrator that affects food intake, gastrointestinal function, and inflammatory responses. NPY/AgRP signaling reduces the expression of CRH, whereas α-MSH signaling increases its expression (61). Central administration of CRH results in decreased food intake and anorexia (62).

Wang et al. (63) reported that the signals induced by EA stimulation at the gastric shu (BL21) and Zhongwan (CV12) in rats are concentrated in the hypothalamic PVN and the dorsal vagal complex (DVC), and increasing gastrointestinal hormones in the PVN and gastral cavity contributes to the regulation of gastric motility. Ovariectomy in rats leads to an increase in food intake and body weight, and a decrease in CRH levels and energy metabolism of the PVN (64). Reports by Zhao et al. (65, 66) show that EA stimulation at CV4, Zhongji (CV3), and Zigongxue (EXTRA22) significantly upregulates the expression of hypothalamic CRH in ovariectomized rats. Moreover, Li et al. (67) have reported that EA stimulation activates the CRH neurons and promotes the secretion of CRH in the PVN of the hypothalamus to alleviate inflammatory responses. Current studies in this field are mainly focused on metabolic disturbance and inflammation, and most of the experimental models are ovariectomized. It has been proved that ovariectomy can increase food intake and body weight, and reduce the content of CRH in the hypothalamus. In summary, these studies indicate that upregulation of CRH in the PVN may play vital roles on weight reduction, and additional studies on acupuncture are needed to confirm this assumption.

## Ventromedial Hypothalamic Nucleus (VMH)

The VMH, classically viewed as the “satiety center”, mainly receives projections of NPY/AgRP and POMC neurons from the ARC and projects its axons to other hypothalamus nuclei and the brainstem. The VMH plays a vital role in energy metabolism and food intake. Lesions of the VMH have been shown to lead to hyperphagia and obesity, whereas the electrical stimulation of VMH results in decreased food intake (68, 69).

Asamoto et al. (70) have shown that stimulation of specific auricular acupuncture points in obese rats effectively activates the satiety center that evokes potentials in the VMH, thus reducing body weight. A similar finding in obese rats has been reported by Shiraishi (71). Liu et al. (72) showed that the excitability of the VMH in obese rats is lower, while acupuncture can enhance the excitability and effectively reduce food intake, eventually leading to weight loss. Zhao et al. (73) further observed the time-effect of acupuncture on obesity and reported that the application of EA at ST36 and Neiting (ST44) leads to increased electrical activity of VMH in obese rats, and results in long-term effect of satiety. Taken together, these findings support that acupuncture can effectively enhance the excitability of VMH, reduce food intake, and ultimately control body weight of animal models.

Brain-derived neurotrophic factor (BDNF), a protein that contributes to neural plasticity, is an anorexigenic signal that is abundantly expressed in the VMH. BDNF is a vital downstream effector of the melanocortin system and its expression is largely upregulated by the activation of melanocortin (74). BDNF in the VMH decreases food intake and body weight, whereas the overexpression of BDNF in the hypothalamus promotes the browning of white adipose tissue through sympathetic activity, thus increasing energy expenditure (75). On the other hand, the deletion of the BDNF gene in the VMH results in an increase in food intake and culminates in obesity (76). The expression of BDNF is negatively correlated with body weight and BMI, which is reduced in patients with obesity and metabolic syndrome (77). Therefore, increasing the expression of BDNF may have a favorable effect on improving metabolic function and obesity. Novel interventions focused on BDNF are being developed for obesity and related metabolic disorders.

Walsh et al. (78) report that exercise training increases the expression of BDNF in obese rats, which consequently improves metabolic function and reverses obesity. Sun et al. (79) have reported that EA at Baihui (DU20) and Shenting (DU24) can increase BDNF levels in patients, which are positively correlated with an improvement in cognition. Additionally, studies have also suggested that obesity can result in cognitive dysfunction (80). Taken together, these findings only support that the elevated expression of BDNF in VMH may be beneficial for ameliorating obesity. However, the underlying mechanisms of acupuncture on BDNF in VMH require validation in future studies.

## Dorsomedial Hypothalamic Nucleus (DMH)

The DMH, located in the dorsal side of the VMH, has widespread links with other hypothalamus nuclei (such as the ARC and PVN) and receives projections of the NPY/AgRP neurons. As with the other sites of the hypothalamus, the microinjection of various orexigenic signals in DMH stimulates food intake. The expression of NPY is highly induced in the DMH of obese mice, and the metabolic efficiency decreases with an increase in food intake and body weight (81, 82). Meanwhile, DMH may collaborate with the PVN, and contributes to maintaining food intake and satiety, while its inhibition leads to hyperphagia and obesity (83).

A study by Golanov et al. (84) suggests that the destruction of the DMH in rabbits results in blocking the inhibition of electroacupuncture stimulation by the evoked potentials in the central thalamus. Moreover, Huang et al. (85) have reported that after EA stimulation, the rate of spontaneous discharges and pain-evoked discharges in the pain-excitation units in the DMH of rats was notably reduced, suggesting that EA therapy can modulate the pain-sensitive neurons of DMH and is involved in the process of acupuncture analgesia. Nevertheless, the effect of acupuncture on DMH, feeding, and energy metabolism, as well as the mechanism of acupuncture on DMH in improving obesity remains to be further elucidated. Additional pathways in future studies remain to be further explored.

## Lateral Hypothalamic Area (LHA)

The LHA, broadly viewed as the “feeding center”, receives neuronal projections from the ARC and is a central regulator associated with feeding behavior and energy balance. LHA is an inseparable component of the appetite-regulatory network. Lesions of the LHA result in reduced appetite and weight loss, whereas stimulation of the LHA has been associated with hyperphagia and obesity (86, 87). Orexigenic neuropeptides, such as orexins and melanin-concentrating hormone (MCH), are present in the LHA. NPY, AgRP, and α-MSH have been detected in the LHA and are closely related to MCH and orexins (88). Both MCH and orexin neurons are involved in the regulation of food intake and energy homeostasis (89), but their mechanisms of acupuncture on obesity remain to be elucidated.

Liu et al. (90) have reported that the critical factor of acupuncture on the treatment of obesity may be through the effective regulation of LHA in obese rats. Meanwhile, Shiraishi et al. (71) reported that stimulation of auricular acupuncture inhibited the excitability of LHA in obese rats *via* a branch of the vagus nerve, which resulted in the feeling of satiety. Additionally, Ma et al. (91) have reported that EA at ST36 and ST44 can effectively suppress gastric hyperactivity induced by the excitation of LHA in rabbits, thus suppressing appetite, relieving hunger, and reducing body weight. Although subjects in these studies were different and acupuncture treatment varied, they all reached a consistent conclusion. In summary, these findings support that acupuncture has an inhibitory effect on the activity of the LHA neurons and can be helpful to achieve weight-loss goals, the specific pathways and mechanisms need to be further explored.

## Interactions Between Hypothalamus and Brainstem

Hypothalamic nuclei and corresponding neuropeptides exert their effects on food intake and energy balance. Generally speaking, the hypothalamus is considered as the pivot of appetite signals, receiving afferent signals from the brain stem and periphery. The brainstem plays a key role in integrating signals from peripheral to central and regulating the equilibrium of energy metabolism (92). And there exist extensive appetite circuits between the hypothalamus and the brainstem, especially the nucleus of the tractus solitaries (NTS).

Similar to the ARC, the NTS is roughly close to a circumventricular organ of semipermeable blood–brain barrier, i.e., the area postrema of the brainstem. Therefore, not does the NTS receive peripheral circulation signals but can also receive vagal afferents from the gastrointestinal tract. Results from gene expression analysis indicated that the transcriptome changes are mostly concentrated in DMH and NTS, suggesting that DMH and NTS play crucial roles in regulating body weight (93). In addition, the NTS in the medulla also contains a large number of POMC neurons related to energy homeostasis.

EA at ST36 upregulated the expression of POMC in the NTS of obese rats, suppressed food intake and reduced body weight (50). It also confirmed that NTS received neuron projections from the hypothalamus. In a study comparing the effects of different EA combinations on NTS neurons, Fang et al. (94) found that 2 Hz EA at ST36 exerted the strongest excitatory effect on NTS neurons, while 100 Hz EA at ST25 exerted the strongest inhibitory effect on NTS neurons in normal rats. These results suggest that different effects of EA on NTS neurons in normal rats may be impacted by acupoint combinations, and EA frequencies also play a significant role. Additional pathways of acupuncture acting on NTS neurons need to be investigated in future studies.

## Access of Peripheral Signals to the Hypothalamus

Owing to the semipermeable blood–brain barrier, peripheral signals, such as leptin, insulin and ghrelin, rapidly gain access to the hypothalamus and are involved in the regulation of the hypothalamic neurons that control food intake and energy expenditure. Additionally, CCK is accessible to the NTS via the vagus nerve and is probably connected to the hypothalamus, interfering with food intake and energy consumption.

## Leptin

With the discovery of leptin, the regulatory role of neuropeptides in food intake and body weight has been better understood. Leptin crosses the blood–brain barrier mainly through a saturable transporter system and exerts its anorexia effect on the hypothalamic regions *via* its receptor. Both POMC/CART and NPY/AgRP neurons in the ARC express leptin receptors, indicating that these effects are mediated by the hypothalamus. Leptin, a product of the ob gene, is the peripheral satiety signal secreted by the adipose tissue. A mutation of the ob gene leads to a deficiency of the circulating leptin, resulting in the obese phenotype in mice, which can be normalized by the administration of leptin (95). Similarly, mutations can cause a deficiency of leptin in humans, leading to increased appetite and resulting in severe obesity, thereby suggesting that leptin plays an important role in maintaining energy metabolism (96). It has been well-documented that leptin exerts an inhibitory effect on food intake and increases energy consumption *via* suppressing the NPY/AgRP neurons and activating α-MSH/CART neurons in the hypothalamus, thus effectively reversing obesity (97, 98).

Sibutramine is mainly used to enhance the sense of satiety, thereby reducing food intake and procuring weight loss (99). When acupuncture or sibutramine was used in obese rats, the reduction of serum leptin in the acupuncture group was more pronounced than that in the sibutramine group (100), indicating that acupuncture is more conducive to regulating serum leptin levels. Similar results in obese patients have also been confirmed by Kang (101). Kim et al. (102) have shown that EA stimulation at ST36 for 4 weeks increased the levels of serum leptin in rats. On the contrary, EA was shown to decrease leptin levels along with weight reduction in obese rats (103–105). Similar findings in obese patients have been reported by Cabioğlu, Luo, Güçel, and Darbandi (106–109). Interestingly, Hsu et al. (110) have reported that auricular acupuncture can reduce the expression of leptin without alleviating body weight in obese patients, which may be attributed to the therapy and small sample. And the efficacy of auricular acupuncture on weight reduction merits further exploration. Moreover, 2-Hz EA treatment in rats with polycystic ovary syndrome has been shown to restore the expression of leptin in visceral adipose, but does not affect serum leptin concentrations (111). These findings indicate that the effect of acupuncture on regulating leptin level depends on the state of energy balance in animals.

As obesity is characterized by leptin resistance and hyperleptinemia, the downregulation of leptin induced by acupuncture may be beneficial in the regulation of leptin in obese models, and more importantly, the intrinsic sensitivity of subjects to leptin may be a key factor in determining the incidence of obesity. Therefore, correcting leptin resistance is essential for the reversal of obesity. Gong et al. (112) have shown that EA treatment significantly reduces serum-leptin levels and increases the expression of leptin receptor mRNA, implying that improving EA-induced leptin sensitivity may have helped reduce the body weight in obese rats. Similar findings in obese patients have also been reported by Wang (113). In summary, these findings suggest that the reversal of leptin sensitivity and reduction of leptin levels in obese experimental models and subjects may be one of the mechanisms of acupuncture in weight loss. And the actions of serum leptin on the hypothalamus in obese models remain to be explored.

## Insulin

Insulin, an important metabolic hormone secreted by the β-cells of the pancreas is an adiposity signal and regulates blood glucose levels. Similar to leptin, insulin is transported across the blood–brain barrier and is mainly regulated by a saturation process involving the insulin receptors in the microvessels of the brain. Meanwhile, insulin binds to its receptors and enters the brain from the neuronal circuit of the hypothalamus, which is especially adequately expressed in the ARC, and exerts its anorexigenic effect and reduces body weight (114, 115). The levels of insulin are primarily determined by peripheral insulin sensitivity, which is associated with fat distribution and systemic fat storage. Excessive accumulation of adipose tissue is often accompanied by insulin resistance, which is closely related to obesity. Both NPY/AgRP and POMC are important downstream targets of insulin, and one of the core roles of insulin is to produce anorexia by suppressing the NPY/AgRP neurons and stimulating POMC neurons (116). The deletion of the insulin receptor is characterized by hyperphagia and obesity, while ICV administration of insulin decreases food intake and body weight (117).

Of note, growing evidence indicate that acupuncture can regulate the levels of insulin in obese rats (103, 104). Similar findings in obese patients have also been reported by Cabioğlu, Gao, and Güçel (106, 108, 118, 119). Cabioğlu et al. (118) have shown that 2-Hz EA at the ear points (Hunger and Shen Men) and body points (LI4, LI11, ST36 and ST44) reduces blood glucose levels and the body weight in obese patients by increasing the levels of serum insulin and C peptide compared to those in the placebo group. Interestingly, studies have shown that EA at ST36, SP6, CV4, and CV12 decreases the levels of serum insulin and reduces body weight in obese rats, and that EA of 100 Hz is more effective than 30 Hz in reversing obesity (103, 104). Substantial studies support the effect of acupuncture on weight loss by downregulating the levels of serum insulin in obese patients (108, 113, 119). The benign regulation of acupuncture on serum insulin is one of the mechanisms of acupuncture in weight loss. Differences in the results among the studies described above may be attributed to the frequency of EA and acupoint selection.

However, many obese patients develop insulin resistance, owing to the inability of insulin to regulate the metabolism of peripheral tissues. Insulin sensitivity, measured using a hyperinsulinemic or euglycemic clamp, has been shown to be regulated effectively by acupuncture therapy (120–122). Chang et al. (120) have reported that EA of 15 Hz on bilateral ST36 improved glucose tolerance and insulin sensitivity in rats. Meanwhile, Liang et al. (123) have reported that low-frequency EA at ST36 and CV4 ameliorates insulin sensitivity *via* increasing the SIRT1/PGC-1α in skeletal muscle of obese and diabetic mice. It is reported that EA improved insulin resistance in obese rats by reducing the inflammatory responses (124, 125). Wang et al. (113) found that EA can downregulate the level of serum insulin and ameliorate insulin resistance, which led to a weight loss in obese patients. Additionally, mounting evidence supports that acupuncture has been an effective method in improving insulin resistance (126, 127).

In summary, these findings support that the mechanism of acupuncture on weight loss may be owing to the reduction of insulin and amelioration of insulin sensitivity in humans and animal models. And the correlations between insulin and peripheral hormones, as well as hypothalamus in obese models remain to be verified.

## Ghrelin

Ghrelin is a 28 amino acid brain-gut peptide secreted by the endocrine cells of the gastric fundus. It is a peripheral hormone that stimulates the appetite center of the hypothalamus and acts as an orexigenic peptide by increasing food intake, resulting in an increase in body weight (128). Although ghrelin is produced peripherally, it is still partially bound to the axon terminals of the NPY/AgRP neurons in the hypothalamus and activates the expression of NPY and AgRP, suggesting that the hormone may exert its effects on energy metabolism and appetite regulation through its interactions with these neuropeptides (129, 130). Interestingly, the levels of fasting plasma ghrelin in obese individuals and experimental animals have been reported to be significantly lower than those in age-matched lean individuals and shown to be negatively correlated with BMI (131–133). Meanwhile, the downregulation of plasma ghrelin levels in obese individuals may be attributed to elevated leptin or insulin levels, because this peptide is also negatively correlated with fasting plasma leptin and insulin levels (134). Moreover, a study suggests that the downregulation of ghrelin results in circadian disturbances in obese mice (135). Levels of ghrelin are increased preprandially and reduced postprandially, implying that ghrelin exerts crucial action in initiating food intake (136). In addition to food cravings, ghrelin can also increase the frequency of meals and reduce the incubation period of feeding (137). Thus, hyperphagia induced by ghrelin was considered to be involved in the formation and aggravation of overweight or obesity.

Wang et al. (138) have mentioned that 2-Hz EA can effectively reduce body weight and partially restore the circadian rhythm in obese rats resulting from ghrelin downregulation. Hsu et al. (110) have reported that ghrelin levels are increased after 6 weeks of auricular acupuncture, whereas those of leptin are decreased without weight reduction in obese women. A study by Güçel et al. (108) indicates a significant upregulation of ghrelin after 5 weeks of acupuncture in obese subjects. Acupuncture may increase the expression of peripheral ghrelin while enhancing satiety. Since the regulation of ghrelin is impaired in obesity patients, it is reasonably speculated that related neuropeptides or hormones may compensate for the loss of ghrelin function. And ghrelin may antagonize the effects of anorexic peptides through the vagus nerve. Therefore, the modulation on ghrelin achieved by acupuncture may play a minor or indirect role in improving obesity. And the effect of acupuncture on ghrelin in obesity remains to be verified.

## CCK

Cholecystokinin (CCK), originates from the gastrointestinal (GI) tract, is transmitted from peripheral to the NTS *via* the vagus nerve. Satiety signal of CCK in NTS may integrate with the hypothalamic inputs implicated in food regulation, and its activation in the NTS results in decreased food intake and weight loss and eventually results in anorexia (139, 140). CCK has been known to be a key satiety signal and exert inhibitory actions on food intake in rodents (141). It is reported that plasma CCK levels were markedly lower in high-fat-fed prone (OP) rats than that of obesity-resistant rats after lipid gavage, implying that the decreased or deficit levels of CCK in OP rats might prone to weight gain and obesity (142).

Kim et al. (143) reported that EA at ST36 markedly enhances satiety *via* the endogenous pathway of CCK in rats. Moreover, EA at ST36, ST40, CV12 and CV4 can suppress appetite and reinforce insulin sensitivity in obese rats, which may be attributed to the elevated expression of serum CCK level (144). Wang et al. (145) compared the effects of EA at different acupoints selection on leptin and CCK in obese rats. Their study demonstrated that there was no differences between the two acupoint selections in reducing body weight. Both EA at the abdominal acupoints (ST25, CV4 and CV12) and lower limbs acupoints (ST36 and ST40) can facilitate weight reduction in obese rats, which is relevant to the increased expression of serum CCK and decreased expression of serum leptin. In summary, these findings suggest that the upregulation of CCK may be the mechanism of acupuncture that results in the inhibition of food intake in experimental models. However, the relationship between CCK and peripheral hormones, and the specific mechanisms of acupuncture acts on the CNS *via* CCK remains to be explored.

## Discussion

**Figure 1:**
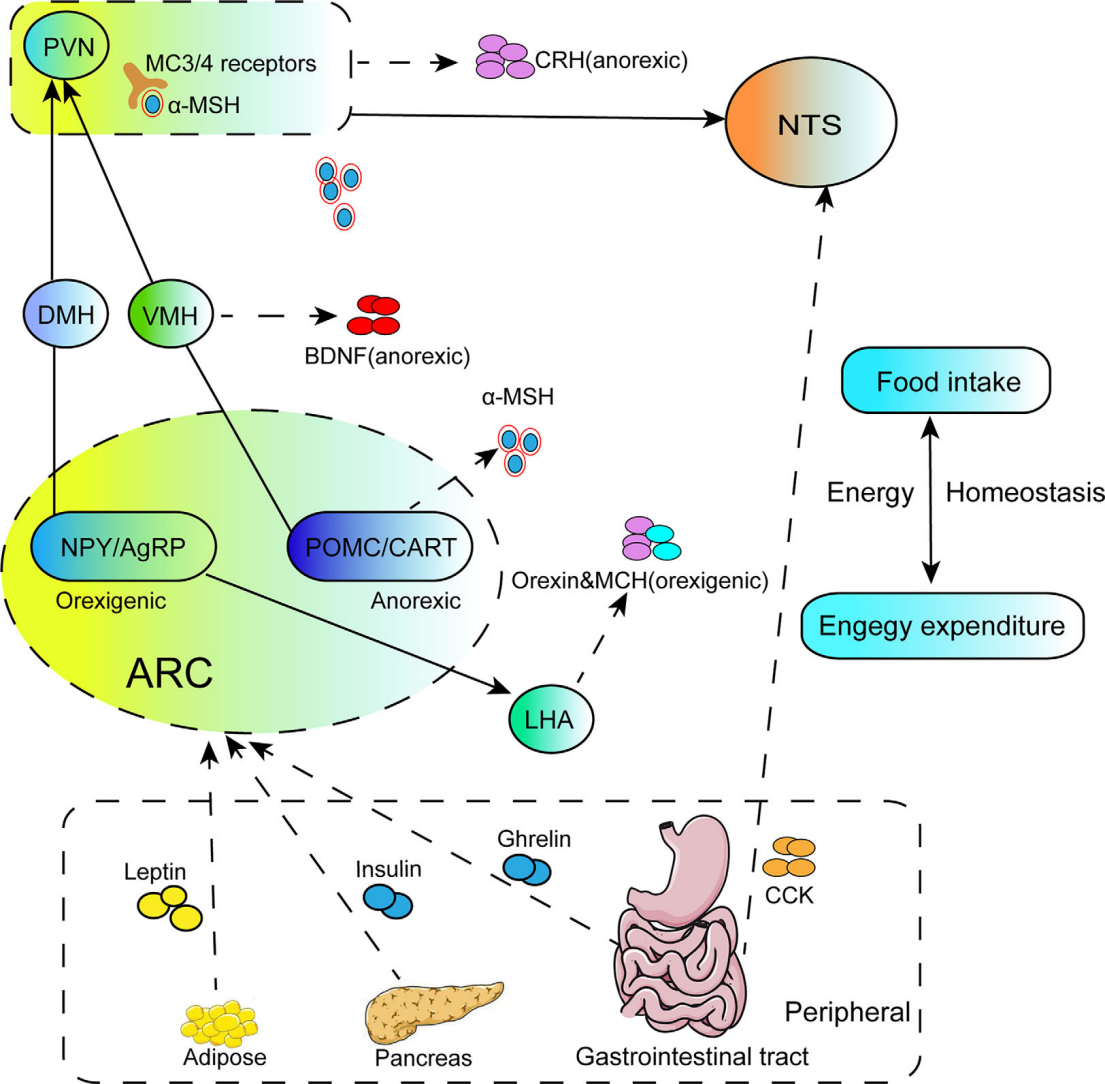
The illustration focuses on the regulation of energy homeostasis by the hypothalamus. NPY/AgRP and POMC/CART neurons located in the ARC of hypothalamus project to secondary neurons such as PVN, DMH, VMH, LHA, and secrete orexigenic AgRP or anorectic POMC. The MC3/4R in PVN are activated after binding with the active component a-MSH cleaved by POMC, and the integrated signals are transmitted to the NTS to regulate feeding behavior. Meanwhile, PVN also secretes anorexic neurohormones CRH to suppress food intake. BDNF located in the VMH is the downstream effector of a-MSH, which plays a role in inhibiting food intake. LHA is mainly stimulated by NPY/AgRP neurons in the ARC and secretes orexigenic peptides, orexin and MCH. Peripheral signals from adipose tissue, pancreas or the gastrointestinal tract either enter the hypothalamus through the median eminence, or connect to the NTS in the brainstem via acting on the vagus nerve afferents, and these signals effectively induce activities of the related nuclei, such as ARC and NTS, then influence the second order neurons of PVN, VMH. DMH and LHA.

Obesity is a common metabolic disorder disease characterized by increased food intake and decreased energy consumption. Due to the complexity of its pathological process, it involves the connections between multiple brain regions. Therefore, deepening understanding of the pathological basis of obesity may provide new insights for the treatment of obesity. It is well-established that hypothalamus plays pivotal role in the regulation of food intake and energy homeostasis. Meanwhile, the hypothalamic nuclei project to other brain regions, such as NTS, and also receive input from NTS and peripheral hormones. The hypothalamic nuclei, neuropeptides, NTS and peripheral signals closely interact to form a complicated appetite-regulation loop (**Figure 1**). The review briefly introduces these mechanisms of hypothalamic neural circuits underlying appetite and energy homeostasis, and probes potential targets for the treatment of obesity.

**Figure 2:**
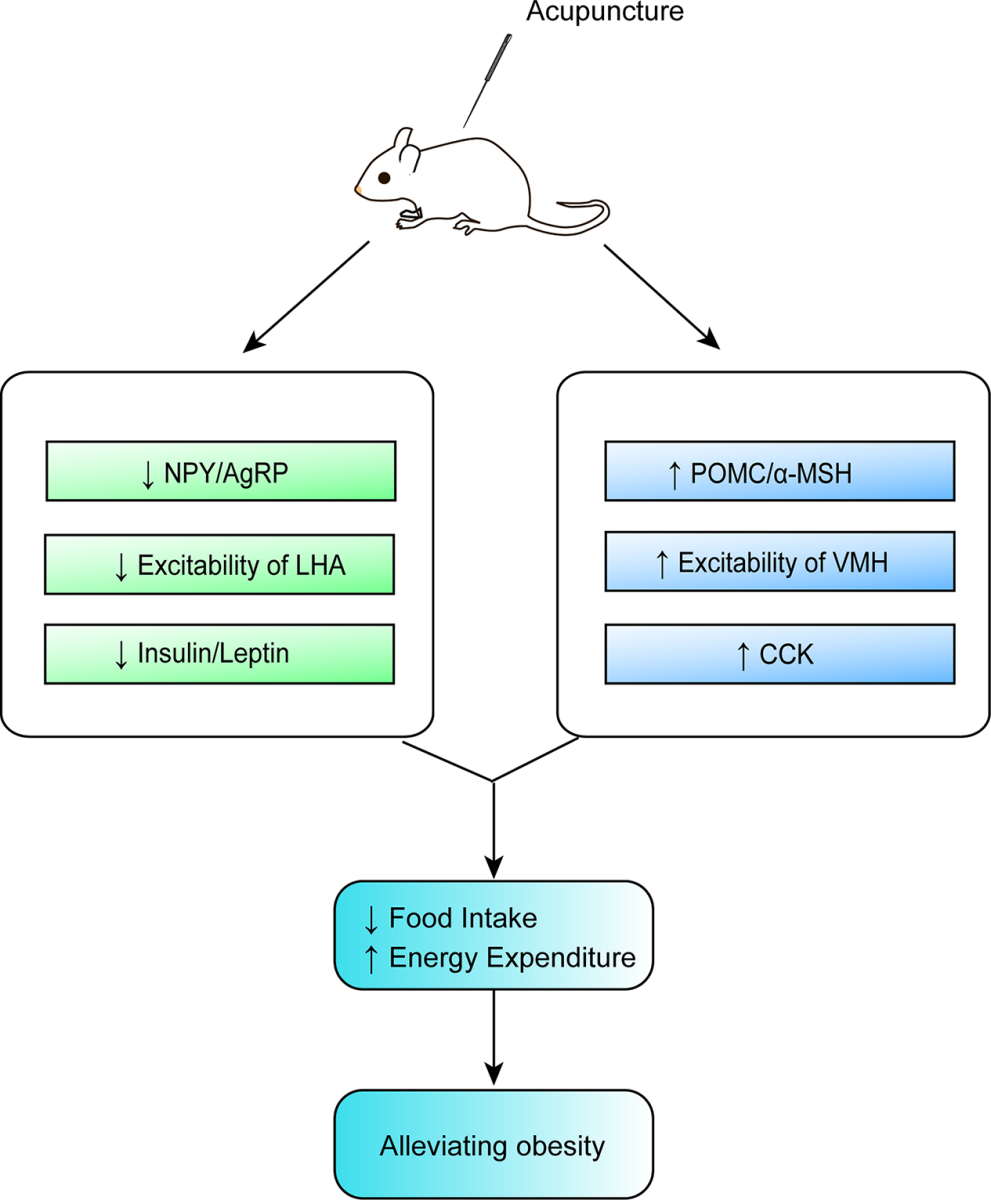
Mechanisms of action of acupuncture in obesity.

Although acupuncture can ameliorate obesity, the molecular biological mechanisms involved in its beneficial effect remain unclear. From this updated review, we conclude that the possible mechanisms of action of acupuncture in obesity involve the upregulation and an increase in the expression of POMC and α-MSH in the ARC, a reduction in the expression of NPY and AgRP in the ARC, an enhancement of the excitation of VMH neurons and the inhibition of the activity of LHA neurons, a decrease in the expression of leptin and insulin, an improvement in the sensitivity of leptin and insulin, as well as an increase in the expression of CCK, all of which provide new insights in the prevention and treatment of obesity by acupuncture. These findings shed light on the complex mechanism of acupuncture in the regulation of appetite from the perspective of hypothalamus, yet the molecular mechanism of its actions on weight control is partially understood (**Table 1** and **Figure 2**). Previous studies on the improvement of obesity by acupuncture were mostly limited to a certain nucleus, while existing studies are more targeted. According to the current findings, it is known that the pivotal targets of acupuncture for improving obesity are mainly focused on the neurons or neuropeptides in the hypothalamic ARC, the peripheral hormones (leptin and insulin). **Figure 1** illustrates that the ARC neurons of the hypothalamus secrete orexigenic/anorexic substances and project them downstream to the hypothalamic nuclei and extrahypothalamic areas involved in the regulation of food intake. Furthermore, this updated review has also pointed out that the prime neuropeptides or proteins induced by acupuncture in regulating food intake are mainly concentrated in the ARC of the hypothalamus. Overall, it can thus be concluded that one of the main mesoscopic targets of acupuncture in ameliorating obesity is located in the hypothalamic ARC. However, the underlying mechanisms for this beneficial effect remain unclear. And it would be interesting to validate the actions of acupuncture on gut-brain axis or peripheral-central feeding in obesity.

**Table 1:** Characteristics of acupuncture in the treatment of obesity derive from main studies included.

Reference	Model	Intervention type	Acupoint	Frequency (duration)	Effect
Kim et al. (36)	SD rats	AAS	Stomach point	twice per day (2 days)	↓NPY
Tian et al. (38)	SD rats	EA	Zusanli(ST36),Sanyinjiao(SP6)	once every other day (NR)	↓NPY, ↓ghrelin, ↓food intake, ↓BW
Shu et al. (39)	Wistar rats	EA	Zusanli(ST36), Guanyuan(CV4), Zhongwan (CV12), Fenglong (ST40)	3 per week (8 wks)	↑SIRT1, ↓NPY, ↑POMC, ↓Ac-FOXO1,↑insulin sensitivity
Liu et al. (42)	SD rats	EA	Zusanli(ST36), Quchi(LI11)	7 per week (4 wks)	↓NPY, ↓AgRP
Ren et al. (43)	Wistar rats	EA	Zusanli(ST36), Guanyuan(CV4), Zhongwan (CV12), Fenglong (ST40)	3 per week (8 wks)	↑POMC, ↓AgRP, ↓food intake, ↓BW
Tian et al. (47)	SD rats	EA	Zusanli(ST36),Sanyinjiao(SP6)	3 per week (4 wks)	↑POMC,↑α-MSH, ↓food intake, ↓BW
Wang et al. (49)	SD rats	EA	Zusanli(ST36),Sanyinjiao(SP6)	7 per week (2 wks)	↑α-MSH, ↓food intake, ↓BW
Ji et al. (50)	Obese rats	EA	Zusanli(ST36)	twice a day (1 wk)	↑POMC, ↑nNOS, ↑ TRPV1, ↓food intake, ↓BW
Huang et al. (51)	Wistar rats	EA	Zusanli(ST36), Guanyuan(CV4), Zhongwan (CV12), Fenglong (ST40)	3 per week (8 wks)	↑POMC, ↑SIRT1, ↓blood lipids, ↓PPG, ↓food intake, ↓BW
Tian et al. (56)	SD rats	EA	Zusanli(ST36),Sanyinjiao(SP6)	3 per week (4 wks)	↑CART, ↓food intake, ↓BW
Leng et al. (57)	SD rats	EA	Zusanli (ST36),Tianshu (ST25), Zhongwan(CV12),Sanyinjiao(SP6)	5 per week (4 wks)	↓methylation of Tsc1, ↓mTORC1, ↑POMC, ↓AgRP, ↓NPY, ↓food intake, ↓BW
Wang et al. (63)	SD rats	EA	Zhongwan(CV12),gastric shu (BL21)	7 per week (1 wk)	↑MTL, ↑GAS
Asamoto and Takeshige (70)	Wistar rats	AAS	Stomach(CO4), Endocrine(CO18),Heart(CO15),Lung(CO14)	NR	↑excitatory of VMH
Shiraishi et al. (71)	Wistar rats	AAS	NR	NR	↑excitatory of VMH, ↓excitatory of LHA
Liu et al. (72)	SD rats	EA	Zusanli (ST 36), Neiting (ST 44)	3 per week (2 wks)	↑excitatory of VMH, ↓DA, ↑5-HT,↓BW
Zhao et al. (73)	SD rats	EA	Zusanli (ST 36), Neiting (ST 44)	(2 wks)	↑excitatory of VMH
Wang et al. (100)	SD rats	EA	Zusanli(ST36), Guanyuan(CV4), Zhongwan(CV12), Sanyinjiao(SP6)	7 per week (4 wks)	↓leptin, ↓TG, ↓BW, ↓insulin
Kang et al. (101)	Obese patients	EA plus AAS	Zusanli(ST36),Guanyuan(CV4),Tianshu(ST25), Sanyinjiao(SP6), Fenglong (ST40); Shenmen(TF4), Spleen(CO13), Stomach(CO4), LargeIntestine(CO7), Endocrine(CO18), Sanjiao(CO17)	3 per week (4 wks)	↓leptin
Ge et al. (103)	SD rats	EA	Zusanli(ST36), Guanyuan(CV4), Zhongwan(CV12), Sanyinjiao(SP6)	7 per week (4 wks)	↓leptin, ↓TG, ↓insulin
Wang et al. (104)	SD rats	EA	Zusanli(ST36), Guanyuan(CV4), Sanyinjiao(SP6), Zhongwan(CV12)	7 per week (4 wks)	↓leptin, ↓insulin, ↓TG, ↓TC, ↓LDL-C
Yan et al. (105)	Wistar rats	EA	Zusanli(ST36), Zhongwan(CV12)	7 per week (4 wks)	↓leptin, ↓BW
Kim et al. (102)	SD rats	EA	Zusanli(ST36)	7 per week (4 wks)	↑leptin, ↓BW, ↓food intake
Cabioğlu and Ergene (106)	Obese patients	EA plus AAS	Hungry point, Shen Men(TF4), Hegu (LI4), Quchi (LI 11), Tianshu (ST25), Zusanli (ST 36), Neiting (ST44)	7 per week (3 wks)	↓leptin, ↓BW
Luo and Li (107)	Obese patients	EA	Zusanli(ST36), Tianshu (ST25), Zhongwan (CV12), Fenglong(ST40), Fujie(SP14), Shuifen(CV9)	3-4 per week (8 wks)	↓leptin, ↑adiponectin
Güçel et al. (108)	Obese patients	Acupuncture	Zusanli(ST36), Neiting (ST44), Sanyinjiao(SP6), Hegu(LI4)	2 per week (5 wks)	↓leptin, ↓insulin, ↓BW, ↓BMI, ↑Ghrelin, ↑CCK
Darbandi et al. (109)	Obese patients	EA	Tianshu(ST25), Zhongwan (CV12), Shuifen(CV9), Sanyinjiao(SP6), Guanyuan(CV4), Weidao(GB28)	2 per week (6 wks)	↓leptin
Hsu et al. (110)	Obese women	AAS	Hungry point, Shen Men(TF4), Stomach(CO4), Endocrine(CO18)	2 per week (6 wks)	↓leptin, ↑ghrelin
Gong et al. (112)	SD rats	EA	Zusanli(ST36), Neiting (ST44)	3 per week (4 wks)	↓leptin, ↓BW
Wang et al. (113)	Obese patients	EA	Zhongwan(CV12), Tianshu (ST25), Daheng (SP15), Daimai (GB26), Shuidao(ST28), Zhigou (TE6), Yinlingquan(SP9), Zusanli (ST36), Fenglong (ST40), Sanyinjiao (SP6)	3 per week (10 wks)	↓leptin, ↓insulin, ↓BMI
Cabioğlu and Ergene (118)	Obese patients	EA plus AAS	Hungry point, Shen Men(TF4), Hegu (LI4), Quchi (LI 11), Zusanli (ST 36), Neiting (ST44)	7 per week (3 wks)	↑insulin, ↑C-peptide, ↓glucose
Gao et al. (119)	Obese patients	EA plus AAS	Tianshu(ST25), Guanyuan(CV4), Sanyinjiao(SP6), Fenglong(ST40), Zusanli(ST36); Shenmen(TF4), Spleen(CO13),Stomach(CO4), LargeIntestine(CO7), Endocrine(CO18), Sanjiao(CO17)	7 per week (8 wks)	↓insulin
Liang et al. (123)	C57BL mice	EA	Zusanli(ST36), Guanyuan(CV4)	5 per week (8 wks)	↑SIRT1,↑insulin sensitivity, ↓BW, ↓food intake
Huang et al. (124)	Wistar rats	EA	Zusanli(ST36), Guanyuan(CV4), Zhongwan (CV12), Fenglong (ST40)	3 per week (8 wks)	↓IL-6, ↓TNF-α, ↓IL-1β,↓MCP-1, ↑IL-10, ↓insulin, ↑insulin sensitivity
Huang et al. (125)	Wistar rats	EA	Zusanli(ST36), Guanyuan(CV4), Zhongwan (CV12), Fenglong (ST40)	3 per week (8 wks)	↑SIRT1, ↓Ac-NFκB, ↓IL-6,↓TNF-α, ↓BMI
Kim et al. (143)	SD rats	EA	Zusanli(ST36)	NR	↓food intake
Song et al. (144)	Wistar rats	EA	Fenglong(ST40),Zhongwan(CV12), Guanyuan(CV4), Zusanli(ST36)	3 per week (8 wks)	↑CCK, ↓insulin, ↑insulin sensitivity, ↓food intake, ↓BW
Wang et al. (145)	Wistar rats	EA	Zhongwan(CV12),Guanyuan(CV4),Tianshu(ST25); Zusanli(ST36), Fenglong(ST40)	3 per week (8 wks)	↓leptin, ↑CCK, ↓food intake, ↓BW

BMI, body mass index; BW, body weight; EA, electro‐acupuncture; AAS, auricular acupoint stimulation; ↑, increase; ↓, decrease; SIRT1, sirtuins 1; DA, dopamine; LDL-C, lipid-cholesterol; NR, not reported; wk, week; wks, weeks; TG, triglycerides; TC, total cholesterol; GAS, gastrin; MTL, motilin.

**Figure 3:**
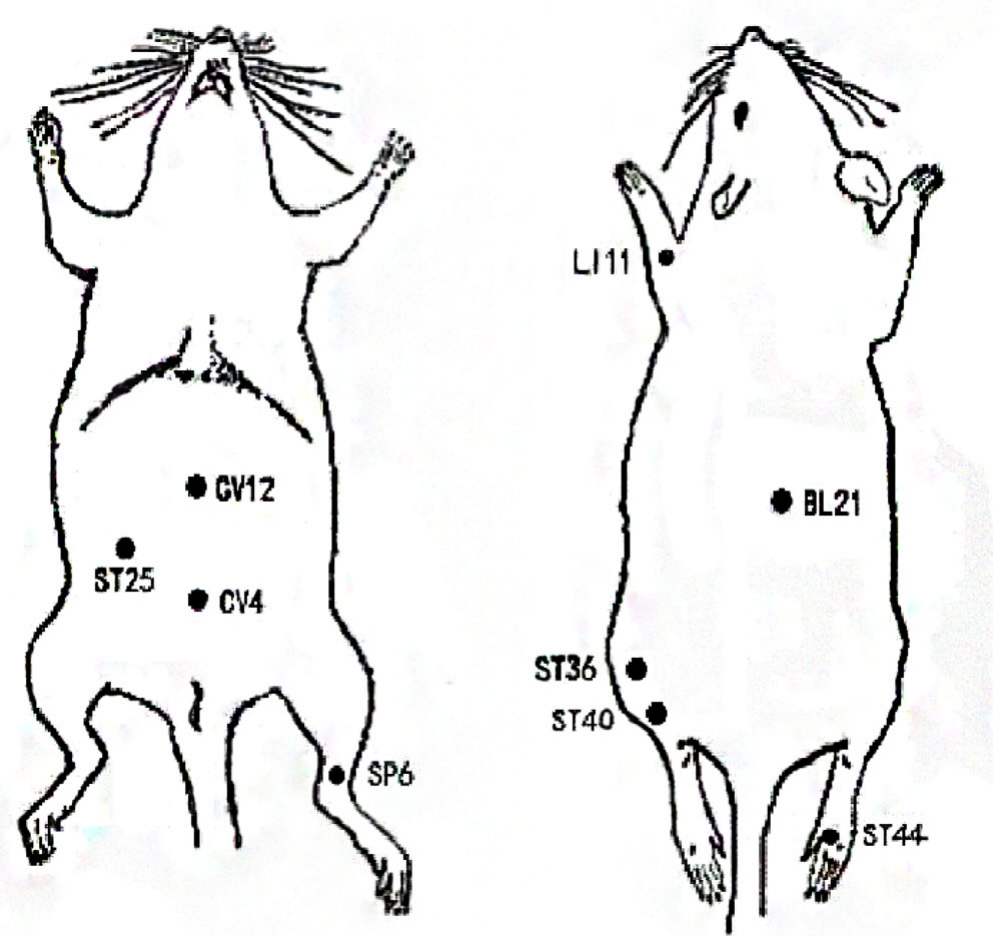
Locations of frequently chosen acupoints in obese rats.

**Figure 4:**
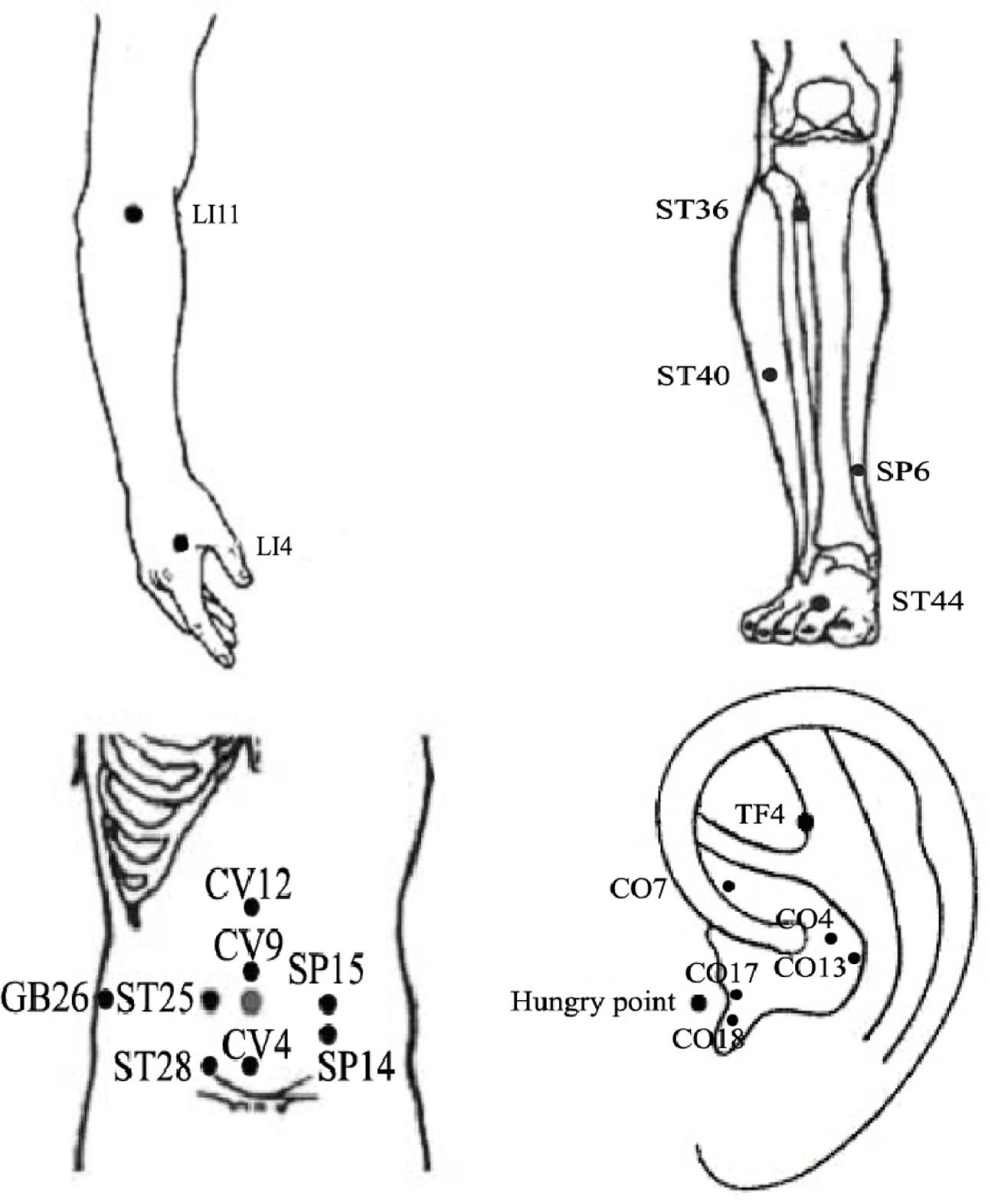
The location of frequently chosen acupoints on humans for the treatment of obesity.

As illustrated in **Figure 2**, acupuncture has the advantages of being used in modulating multiple targets and pathways, although there are still some issues that limit the use and subsequent benefits of this treatment modality. First, current studies targeting on reducing obesity by acupuncture mainly focus on the ARC or a certain nucleus, and rarely involve specific activated neurons, their correlation with various pathophysiologies, and the projections between neurons. Thus, it is crucial to fully unveil the characteristics and projections of these neurons. Owing to the human body is an organic whole, the pathological process of obesity may be owing to the comprehensive action of the target organs and specific neurons and may be consistent with the new idea of neural circuits. New technology such as metabonomics, proteomics, transcriptomics, and neural tracing may help explain the mechanisms of acupuncture on obesity. Second, there are no definite or established standards for EA parameters in the treatment of obesity based on the present evidence. To compare the efficacy of EA using different frequencies in the treatment of obesity, additional large-scale clinical studies or evidence-based assessment should be considered in the future. Moreover, owing to the complicated manipulations involved in acupuncture, including lifting, inserting, twisting, rotating, reinforcing, and reducing, the efficacy of acupuncture may vary based on the different manipulations among acupuncturists, which may weaken existing evidence to some extent. Therefore, standardized acupuncture treatments including the acupoint selection, EA frequency, treatment duration need to be validated. Lastly, the selection of acupoints may significantly influence the reversal of obesity. Owing to the specificity of the acupoints, the compatibility of different acupoints may have different effects and may activate different regions of the brain. Although substantial findings have confirmed the beneficial effects of acupuncture on obese animal models, it remains to be verified in humans. Indeed, the frequently acupoints chosen by acupuncture for the treatment of obesity also differs in rats and humans (**Figures 3** and **4**). And it is of great significance to summary the acupoint prescription for the treatment of obesity. With advances in the standardization of acupuncture, the process of acupuncture and acupoint selection will likely be normalized, resulting in highlighting its use in a clinical setting. In summary, current findings suggest that from the perspective of the hypothalamus, acupuncture can be beneficial in obesity, although its mechanism of action at the molecular level needs to be further elucidated. This aspect will be the focus of our further studies. As mentioned earlier, we further research new technology such as metabonomics, proteomics, transcriptomics, and neural tracing from the perspective of metabolomics which may help explain the mechanisms of acupuncture on obesity.

## Author Contributions 

LW conceived the main ideas and wrote this paper. CCY helped illustrate the figures. JL gave advices and guidance this paper. QT helped search the references. YJD helped revise the manuscript. All authors contributed to the article and approved the submitted version.

## Funding

This work was supported by National Natural Science Foundation of China (No.81873380, No.82074566, No.81473786) and Sun Guojie Inheritance Base for TCM Acupuncture-Moxibustion of World Federation of Acupuncture Moxibustion Societies in Wuhan, China (World Federation of Acupuncture-Moxibustion Societies [2019] No. 26).

## Conflict of Interest

The authors declare that the research was conducted in the absence of any commercial or financial relationships that could be construed as a potential conflict of interest.
